# Nationwide trends in outcomes and resource utilization in surgically treated acute type A aortic dissection with coronary malperfusion

**DOI:** 10.1016/j.xjon.2026.101733

**Published:** 2026-03-19

**Authors:** Kentaro Fukano, Yusuke Sasabuchi, Hiroki Matsui, Yusuke Iizuka, Atsushi Yamaguchi, Masamitsu Sanui, Hideo Yasunaga

**Affiliations:** a Department of Anesthesiology and Critical Care Medicine, Jichi Medical University Saitama Medical Center, Saitama, Japan; b Department of Real-World Evidence, Graduate School of Medicine, The University of Tokyo, Tokyo, Japan; c Department of Clinical Epidemiology and Health Economics, School of Public Health, The University of Tokyo, Tokyo, Japan; d Department of Cardiovascular Surgery, Jichi Medical University Saitama Medical Center, Saitama, Japan; e Division of Intensive Care, Department of Anesthesiology and Intensive Care Medicine, Jichi Medical University School of Medicine, Tochigi, Japan

**Keywords:** acute type A aortic dissection, coronary malperfusion, mechanical circulatory support, administrative database, intensive care unit

## Abstract

**Background:**

Coronary malperfusion (CM) is a life-threatening complication of acute type A aortic dissection (ATAAD) that has been associated with poor outcomes. Although overall surgical outcomes for ATAAD have improved over time, contemporary nationwide trends in mortality and the associated use of intensive care unit (ICU)-level resources among patients with CM remain incompletely characterized.

**Methods:**

We conducted a retrospective cohort study using the Japanese Diagnosis Procedure Combination database from July 2010 to March 2022. Patients who underwent emergency surgery for ATAAD on the day of admission were included. CM was defined by a diagnosis of acute myocardial infarction and/or receipt of coronary angiography and/or percutaneous coronary intervention on the day of admission. Temporal trends in in-hospital mortality were examined, and healthcare resource utilization was compared according to the presence or absence of CM.

**Results:**

Among 31,522 patients with surgically treated ATAAD, 1167 (3.7%) were classified as having CM. In-hospital mortality was substantially higher in patients with CM than in those without CM (34.3% vs 9.6%). After multivariable adjustment, CM was associated with increased in-hospital mortality after multivariable adjustment (adjusted hazard ratio, 1.80; 95% confidence interval, 1.58-2.06). Over the study period, in-hospital mortality declined significantly in patients without CM, whereas no temporal improvement was observed in those with CM. Patients with CM required substantially greater ICU-level resources, including prolonged mechanical ventilation and higher hospitalization costs.

**Conclusions:**

In this nationwide cohort of patients with surgically treated ATAAD, CM was associated with persistently poor short-term outcomes and greater ICU resource utilization, without clear improvement over time.

## Graphical Abstract

**Figure:**
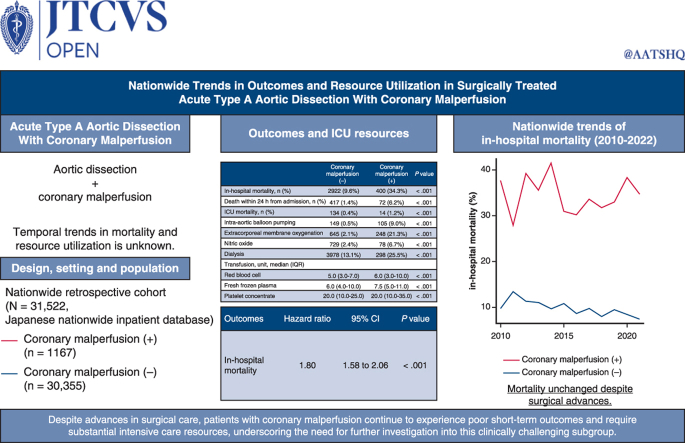

**Figure:**
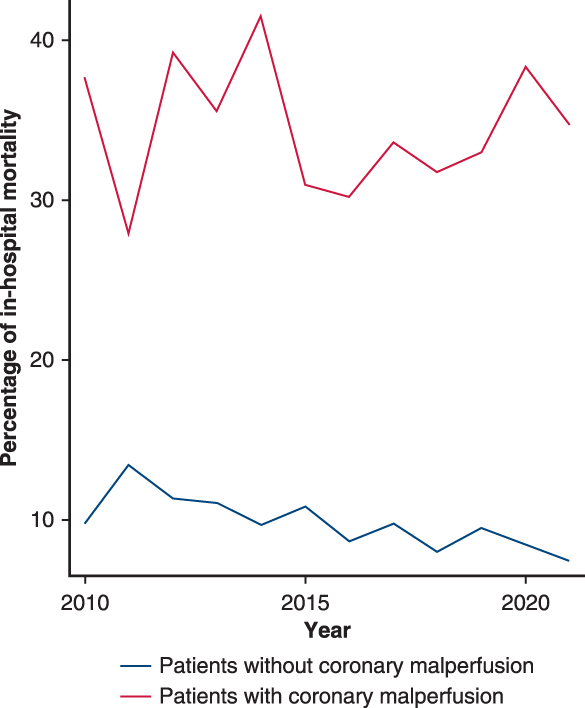
Trends in in-hospital mortality acute type A aortic dissection with coronary malperfusion.

**Central Message**

In this nationwide cohort of surgically treated ATAAD, coronary malperfusion was associated with persistently poor short-term outcomes and greater ICU resource utilization, without clear improvement over time.

**Perspective**

This nationwide study shows that coronary malperfusion in surgically treated acute type A aortic dissection is associated with persistently poor short-term outcomes and high intensive care resource use. By describing long-term outcome patterns at the population level, these findings identify a subgroup with a substantial clinical burden that warrants continued clinical attention and further study.

Acute type A aortic dissection (ATAAD) is a surgical emergency, with mortality increasing rapidly without timely treatment.^1^^,^^2^ Over the past several decades, advances in surgical techniques and perioperative management have contributed to improved outcomes in patients with ATAAD. Data from the International Registry of Acute Aortic Dissection demonstrate a decline in postoperative mortality from 25% in 1995 to 18% in 2013. Additional reductions in 1-year mortality have been reported in more recent cohorts from 2000-2009 and 2010-2019.^3^^,^^4^ These improvements highlight the substantial progress achieved in the overall management of ATAAD.

Despite these advances, malperfusion of vital organs remains a major determinant of poor prognosis in ATAAD. Among these complications, coronary malperfusion (CM) is particularly devastating, as it results in acute myocardial ischemia, severe circulatory instability, and high early mortality.^5^, ^6^, ^7^, ^8^, ^9^, ^10^, ^11^ Previous studies, based largely on single-center experiences or multicenter surgical series, have consistently reported that CM is associated with markedly worse outcomes in patients with ATAAD.^7^^,^^10^, ^11^, ^12^, ^13^ Although CM has been identified as an independent risk factor for mortality in ATAAD, whether the temporal improvements observed in overall ATAAD outcomes have extended to patients with CM remains unknown.^3^^,^^4^ Consequently, population-level evidence evaluating whether improvements in ATAAD outcomes over time have extended to patients with CM remains limited.

Therefore, using a nationwide database, we evaluated temporal trends in in-hospital mortality among surgically treated patients with ATAAD complicated by CM. Here we report our findings and also describe the clinical characteristics, management patterns, and healthcare resource utilization of these patients.

## Methods

### Study Design and Setting

This retrospective cohort study used data from the Japanese Diagnosis Procedure Combination (DPC) database, a nationwide administrative inpatient database. The DPC database includes discharge abstracts and administrative claims data from more than 1000 acute care hospitals, representing approximately 50% of all acute care hospitals and 90% of tertiary emergency centers in Japan.^14^ The database provides detailed patient-level information, including demographics, diagnoses coded according to the International Classification of Diseases, 10th Revision (ICD-10), and treatment-related data. Diagnoses are categorized as primary, admission-precipitating, resource-consuming, preexisting comorbidity, or postadmission complication. The DPC also records procedures, medications, transfusions, and discharge outcomes. Previous validation studies have demonstrated high specificity and moderate sensitivity for diagnosis and high accuracy for procedure coding.^15^ This database has been used to reliably identify aortic dissection cases and evaluate cardiovascular conditions.^16^^,^^17^ Fiscal year information is also recorded and was used to capture temporal changes in practice patterns and reimbursement structures during the study period.

### Ethics

This study was approved by the Institutional Review Board of the University of Tokyo (approval 3501-[5]; approved May 19, 2021). The requirement for informed consent was waived because all data were anonymized. The study was conducted in accordance with the Declaration of Helsinki and is reported according to the STROBE guidelines.

### Inclusion and Exclusion Criteria

Patients admitted between July 2010 and March 2022 were included. The inclusion criteria were as follows: (1) admission-precipitating diagnosis of acute aortic dissection (ICD-10 code I710), (2) emergency admission, and (3) surgical treatment for ATAAD on the day of admission. The eligible procedures included ascending aortic replacement with or without aortic valve surgery, Bentall-de Bono and David procedures, and total arch replacement. Patients were excluded if data on their referral status, ambulance use, consciousness level, transfer from other wards, or body mass index were missing. Patients who did not undergo emergency surgery for ATAAD on the day of admission, including those treated conservatively or with delayed surgery, also were excluded. Accordingly, the present analysis focused on patients with ATAAD who survived to undergo same-day emergency surgery.

### Data Collection and Variables

The covariates included fiscal year, age, sex, body mass index, smoking history, current chemotherapy, Charlson Comorbidity Index,^18^ Barthel Index, and level of consciousness on admission (classified using the Japan Coma Scale).^19^ Preexisting comorbidities included hypertension, dyslipidemia, diabetes, chronic kidney disease, Marfan syndrome, pregnancy, obstructive sleep apnea, bicuspid aortic valve, Loeys-Dietz syndrome, Ehlers-Danlos syndrome, Turner syndrome, immune-mediated vasculitis (eg, Takayasu arteritis, Behçet disease, giant-cell arteritis), tuberculosis, and prior cardiac interventions (eg, valve surgery, coronary artery bypass grafting [CABG], coronary angiography [CAG], percutaneous coronary intervention [PCI]).^20^^,^^21^ Malperfusion was defined as involvement of the cerebral, renal, mesenteric, or iliofemoral territories; the number of affected organs was recorded.

Additional variables on admission included aortic regurgitation, cardiac tamponade, cardiogenic shock, aortic rupture, and spontaneous return of circulation. Admission-related variables included emergency center admission, night/weekend admission, intensive care unit (ICU) or high-dependency unit admission, interhospital transfers, ward transfers, and ambulance transport. The emergency procedures performed on the day of admission included cardiac massage (open or closed chest), emergency intubation, defibrillation, pericardiocentesis, use of vasopressors or inotropes, coronary or aortic surgical management, and volatile anesthesia. Definitions and ICD-10 codes are listed in Online Data Supplement 1. The definitions were based on those used in our previous nationwide analysis of ATAAD treatment strategies, with minor modifications to the current trend analysis.^22^

### Definition of CM

CM was defined operationally using claims-based information because the database does not contain electrocardiographic findings, cardiac biomarkers, or detailed coronary anatomy. Patients were classified as having CM if they had a diagnosis code for acute myocardial infarction (ICD-10: I21.0–I21.4, I21.9–I22.1, I22.8–I23.6, and I24.9), which has shown high specificity in a nationwide claims-based validation study,^17^ and/or receipt of CAG and/or PCI on the day of admission, to capture clinically suspected coronary involvement.^15^

To enhance transparency regarding the operational definition of CM, patients meeting the criteria for CM were further categorized into 3 mutually exclusive subgroups for descriptive purposes: (1) acute myocardial infarction diagnosis only; (2) CAG and/or PCI only; and (3) both acute myocardial infarction diagnosis and CAG and/or PCI (Online Data Supplement 2).

Given the nature of administrative data, we could not distinguish mechanisms of coronary compromise (eg, dynamic compression vs static coronary dissection), determine the persistence of myocardial ischemia, or evaluate indications and procedural details of coronary interventions.

### Outcomes

The primary outcome was the in-hospital mortality rate. Secondary outcomes included 24-hour mortality, ICU mortality, hospital and ICU length of stay (LOS), mechanical ventilation duration, and total hospitalization costs. All costs were converted from Japanese yen to US dollars using the approximate average exchange rate for the study period (1 USD = 110 JPY). Postoperative complications included myocardial infarction, mediastinitis, and postoperative shock (as defined by ICD-10 codes), as well as such postoperative procedures as pulmonary artery catheterization, pacemaker implantation, intra-aortic balloon pump, extracorporeal membrane oxygenation, Impella use, nitric oxide therapy, dialysis, tracheostomy, and transfusion volume.

### Statistical Analysis

Continuous variables are summarized as mean ± standard deviation or median with interquartile range (IQR), as appropriate, and compared using the Student *t* test or Wilcoxon rank-sum test. Categorical variables are expressed as frequency and percentage and compared using the χ^2^ test or Fisher exact test.

To assess the association of CM and in-hospital mortality, we constructed a Cox proportional hazards model and reported hazard ratios (HRs) along with corresponding 95% confidence intervals (CIs). To account for potential within-hospital clustering, robust standard errors clustered at the hospital level were applied for the primary outcome analysis. The proportional hazards assumption was examined using Schoenfeld residuals. For secondary outcomes, including the hospital and ICU LOS and mechanical ventilation, we applied Poisson regression models with robust standard errors to the incidence rate ratios and their 95% CIs. To evaluate the association between CM and total hospitalization costs, we performed a multivariable linear regression analysis. Hospitalization costs were analyzed as descriptive indicators of healthcare resource utilization and not as formal economic evaluations.

All multivariable models were adjusted for covariates selected a priori based on clinical relevance and data availability (Table 1, Online Data Supplement 3). These included variables related to (1) patient demographics and temporal factors (age, sex, year, month), (2) baseline clinical status (body mass index categories, physical function at admission, level of consciousness), (3) comorbidities and past medical history (including Charlson Comorbidity Index, cardiovascular and systemic conditions, and history of chemotherapy), (4) admission characteristics (emergency center admission, admission ward, admission at night or on a weekend, ambulance use, interhospital transfer), (5) acute severity indicators (aortic regurgitation, cardiogenic shock, cardiac tamponade, cardiac arrest/return of spontaneous circulation), (6) malperfusion profiles, (7) procedures and treatments on the day of admission, (8) vasoactive and inotropic drug use on the day of admission, and (9) operative- and anesthesia-related variables (including use of volatile anesthesia).

**Table 1:** Patient characteristics and organ-specific malperfusion of patients with acute type A aortic dissection, stratified by coronary malperfusion status

Characteristic	Total (N = 31,522)	No coronary malperfusion (N = 30,355)	Coronary malperfusion (N = 1167)	*P* value
Age, y, mean (SD)	67.85 (13.04)	67.98 (13.04)	64.50 (12.42)	<.001
Male sex, n (%)	15,276 (48.5)	14,564 (48.0)	712 (61.0)	<.001
BMI, kg/m^2^, n (%)				
<18.5	2795 (8.9)	2720 (9.0)	75 (6.4)	<.001
18.5-24.9	18,753 (59.5)	18,097 (59.6)	656 (56.2)	
25.0-29.9	7755 (24.6)	7425 (24.5)	330 (28.3)	
>29.9	2219 (7.0)	2112 (7.0)	106 (9.1)	
Smoking history (current/past), n (%)	14,743 (46.8)	14,098 (46.4)	645 (55.3)	<.001
CCI, median (IQR)	0.0 (0.0-2.0)	0.0 (0.0-2.0)	0.0 (0.0-2.0)	.88
State of consciousness at admission, n (%)				
Clear	26,857 (85.2)	25,970 (85.6)	887 (76.0)	<.001
Disorientation	1056 (3.4)	1009 (3.3)	47 (4.0)	
Somnolence	1475 (4.7)	1400 (4.6)	75 (6.4)	
Coma	2134 (6.8)	1976 (6.5)	158 (13.5)	
Malperfusion other than coronary artery, n (%)				
0	28,455 (90.3)	27,456 (90.4)	999 (85.6)	<.001
1	2904 (9.2)	2749 (9.1)	155 (13.3)	
2	156 (0.5)	144 (0.5)	12 (1.0)	
3	7 (0.0)	6 (0.0)	1 (0.1)	
Aortic regurgitation at admission, n (%)	3476 (11.0)	3317 (10.9)	159 (13.6)	.004
Cardiac tamponade at admission, n (%)	1110 (3.5)	1059 (3.5)	51 (4.4)	.11
ROSC at admission, n (%)	161 (0.5)	137 (0.5)	24 (2.1)	<.001
Cardiogenic shock at admission, n (%)	761 (2.4)	637 (2.1)	124 (10.6)	<.001
Rupture at admission, n (%)	88 (0.3)	86 (0.3)	2 (0.2)	.48
Introduction from other hospitals, n (%)	16,365 (51.9)	15,861 (52.3)	504 (43.2)	<.001
Procedures on day of admission, n (%)				
Closed chest compression	488 (1.5)	403 (1.3)	85 (7.3)	<.001
Defibrillation	528 (1.7)	431 (1.4)	97 (8.3)	<.001
Open chest compression	50 (0.2)	47 (0.2)	3 (0.3)	.39
Intubation in the ER	3695 (11.7)	3468 (11.4)	227 (19.5)	<.001
Pericardial puncture	484 (1.5)	466 (1.5)	18 (1.5)	.98
CAG	351 (1.1)	0 (0.0)	351 (30.1)	<.001
PCI	166 (0.5)	0 (0.0)	166 (14.2)	<.001
Vasoconstrictors and inotropic drug use on day of admission, n (%)				
Adrenaline	4879 (15.5)	4400 (14.5)	479 (41.0)	<.001
Noradrenaline	24,350 (77.2)	23,322 (76.8)	1028 (88.1)	<.001
Vasopressin	1135 (3.6)	1041 (3.4)	94 (8.1)	<.001
Milrinone	3999 (12.7)	3710 (12.2)	289 (24.8)	<.001
Dopamine	21,600 (68.5)	20,758 (68.4)	842 (72.2)	.007
Dobutamine	20,291 (64.4)	19,381 (63.8)	910 (78.0)	<.001

*SD*, Standard deviation; *BMI*, body mass index; *CCI*, Charlson Comorbidity Index; *IQR*, interquartile range; *ROSC*, return of spontaneous circulation; *ER*, emergency room; *CAG*, coronary angiography; *PCI*, percutaneous coronary intervention.

### Sensitivity Analyses

To assess the robustness of the primary findings to alternative definitions and potential sources of bias, we conducted 2 prespecified sensitivity analyses. First, we repeated the primary analysis using a more specific definition of CM restricted to patients with a diagnosis of acute myocardial infarction only. Second, we performed an analysis limited to patients without interhospital transfer, to minimize potential misclassification related to preadmission management and referral bias.

To describe management patterns among patients with ATAAD complicated by CM (Online Data Supplement 4), we classified individuals into 3 groups according to procedures recorded on the admission day: (1) direct aortic repair without CAG (direct aortic repair group), (2) coronary angiography followed by aortic repair (CAG group), and (3) CAG with PCI before aortic repair (PCI group). These categories were defined for descriptive purposes to summarize contemporaneous practice patterns and were not intended to support causal comparisons regarding the effectiveness of specific strategies.

All statistical analyses and visualizations were performed using Stata/SE 17.0 (StataCorp). Statistical significance was defined as a 2-sided *P* value <.05. Dr Fukano had full access to all study data and is responsible for its integrity and for data analysis. A separate analysis focusing on treatment strategies and associated outcomes using the same dataset has been reported elsewhere.^22^

## Results

### Study Population

**Figure 1:**
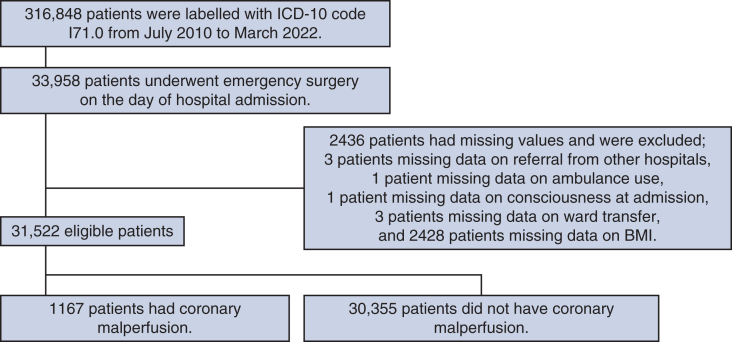
Patient flow in acute type A aortic dissection patients. *ICD-10*, International Classification of Diseases, Tenth Revision; *BMI*, body mass index.

Overall, 316,848 hospitalizations with a diagnosis of ATAAD (ICD-10 code I71.0) were identified in the DPC database during the study period. After restricting the cohort to patients who underwent emergency surgery for ATAAD on the day of admission and excluding 2436 patients with missing key covariates, a total of 31,522 surgically treated ATAAD patients were included (Figure 1). Of these, 1167 (3.7%) were classified as having CM.

### Baseline Characteristics

Baseline characteristics of the patients with ATAAD stratified by the presence or absence of CM are shown in Table 1 and Online Data Supplement 3. The patients with ATAAD and CM were significantly younger than those without CM. The CM group included a higher proportion of male patients and current smokers and had a higher body mass index. At presentation, patients with CM had lower levels of consciousness on admission and were more likely to present with aortic regurgitation, spontaneous return of circulation, and cardiogenic shock. In contrast, interhospital transfer was less common in the CM group. With respect to comorbidities, patients with CM were less likely to have hypertension or dyslipidemia but more likely to have a history of CABG or PCI. On the day of admission, invasive procedures and circulatory support measures, including pulmonary artery catheterization, closed-chest cardiac massage, defibrillation, emergency intubation, CAG, and PCI, were used more frequently in the CM group. The use of vasopressors and inotropic agents was also more common among patients with CM. In terms of surgical procedures, CABG and operations involving the ascending aorta were performed more frequently in patients with ATAAD complicated by CM.

### Temporal Trends in Mortality

**Figure 2:**
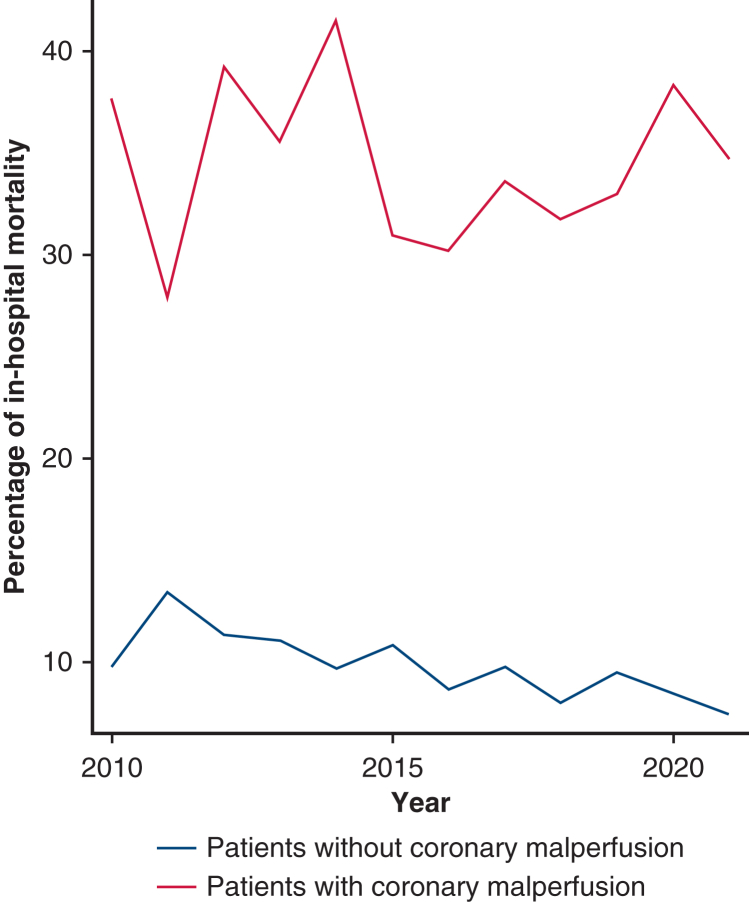
Temporal trends in in-hospital mortality among patients with surgically treated acute type A aortic dissection, stratified by the presence or absence of coronary malperfusion.

Figure 2 shows temporal trends in in-hospital mortality among patients with ATAAD with CM and without CM. Over the study period, in-hospital mortality declined steadily among the patients without CM; in contrast, no corresponding improvement in in-hospital mortality was observed among the patients with CM.

### Primary and Secondary Outcomes

Primary and secondary outcomes are summarized in Table 2, and the results of multivariable analyses are presented in Table 3. In-hospital mortality was significantly higher in the CM group compared to the non-CM group (34.3% vs 9.6%; *P* < .001). Similarly, mortality within 24 hours of admission (6.2% vs 1.4%; *P* < .001) and ICU mortality (1.2% vs 0.4%; *P* < .001) were significantly elevated in the CM group. CM was significantly associated with in-hospital mortality (adjusted HR, 1.80; 95% CI, 1.58-2.06; *P* < .001). Total hospitalization costs were higher in patients with CM (median, 7.37 × 10^4^ USD; IQR, 5.93-9.73 × 10^4^ USD) compared to those without CM (6.12 × 10^4^ USD; IQR, 5.08-7.59 × 10^4^ USD; *P* < .001). CM was significantly associated with total hospitalization costs (difference, 7173 USD; 95% CI, 5205-9141 USD; *P* < .001). Although the median hospital LOS was similar in the 2 groups (24.0 days vs 25.0 days), the median ICU LOS (7.0 vs 6.0 days; *P* < .001) and duration of mechanical ventilation (mean, 13.4 days vs 9.0 days; *P* < .001) were longer in patients with CM. CM was significantly associated with longer total hospital LOS and higher total hospitalization costs.

**Table 2:** Primary and secondary outcomes, postoperative complications, and procedures for the overall cohort and for patients with and without coronary malperfusion among those with acute type A aortic dissection

Parameter	Total (N = 31,522)	No coronary malperfusion (N = 30,355)	Coronary malperfusion (N = 1167)	*P* value
In-hospital mortality, n (%)	3322 (10.5)	2922 (9.6)	400 (34.3)	<.001
Death within 24 h of admission, n (%)	489 (1.6)	417 (1.4)	72 (6.2)	<.001
ICU mortality, n (%)	148 (0.5)	134 (0.4)	14 (1.2)	<.001
Hospitalization costs, 10^4^ USD, median (IQR)	6.15 (5.10-7.65)	6.12 (5.08-7.59)	7.37 (5.93-9.73)	<.001
Hospital LOS, d, median (IQR)	25.0 (17.0-38.0)	25.0 (17.0-38.0)	24.0 (11.0-43.0)	<.001
ICU LOS, d, median (IQR)	6.0 (4.0-10.0)	6.0 (4.0-10.0)	7.0 (4.0-14.0)	<.001
MV duration, d, mean (SD)	9.17 (20.80)	9.01 (20.42)	13.36 (28.68)	<.001
Postoperative complications/procedures, n (%)				
Postoperative myocardial infarction	194 (0.6)	145 (0.5)	49 (4.2)	<.001
Mediastinitis	273 (0.9)	254 (0.8)	19 (1.6)	.004
Postoperative shock	993 (3.2)	942 (3.1)	51 (4.4)	.015
Pulmonary artery catheter insertion	17,014 (54.0)	16,357 (53.9)	657 (56.3)	.10
Pacemaker implantation	786 (2.5)	706 (2.3)	80 (6.9)	<.001
Intra-aortic balloon pumping	254 (0.8)	149 (0.5)	105 (9.0)	<.001
ECMO	893 (2.8)	645 (2.1)	248 (21.3)	<.001
Impella	14 (0.0)	8 (0.0)	6 (0.5)	<.001
Nitric oxide	807 (2.6)	729 (2.4)	78 (6.7)	<.001
Dialysis	4276 (13.6)	3978 (13.1)	298 (25.5)	<.001
Tracheostomy	2225 (7.1)	2111 (7.0)	114 (9.8)	<.001
Transfused units, median (IQR)				
Red blood cell	5.0 (3.0-8.0)	5.0 (3.0-7.0)	6.0 (3.0-10.0)	<.001
Fresh frozen plasma	6.0 (4.0-10.0)	6.0 (4.0-10.0)	7.5 (5.0-11.0)	<.001
Platelet concentrate	20.0 (10.0-30.0)	20.0 (10.0-25.0)	20.0 (10.0-35.0)	<.001

*ICU*, Intensive care unit; *IQR*, interquartile range; *LOS*, length of stay; *MV*, mechanical ventilation; *SD*, standard deviation; *ECMO*, extracorporeal membrane oxygenation.

**Table 3:** Association between coronary malperfusion and primary or secondary outcomes

Outcome	HR	95% CI	*P* value
In-hospital mortality	1.80	1.58-2.06	<.001

	Adjusted IRR	95% CI	*P* value
Hospital LOS	0.82	0.82-0.90	<.001
ICU LOS	1.01	0.95-1.07	.783
Duration of mechanical ventilation	1.04	0.96-1.14	.331

	Difference	95% CI	*P* value
Total hospitalization cost, USD	7173	5205-9141	<.001

*HR*, Hazard ratio; *CI*, confidence interval; *IRR*, incidence rate ratio; *LOS*, length of stay; *ICU*, intensive care unit.

### Postoperative Complications

The incidence of postoperative complications was consistently higher in the CM group (Table 2). Patients in the CM group more frequently experienced postoperative myocardial infarction, shock, and required advanced postoperative interventions. Requirements for blood product transfusion also were greater in patients with CM compared to those without CM.

### Management Patterns in Patients With CM

Trends in management patterns among patients with ATAAD complicated by CM are shown in Online Data Supplement 4. The relative use of direct aortic repair, CAG before aortic repair, and CAG with PCI varied over time, without a consistent temporal trend.

### Sensitivity Analyses

Results of the prespecified sensitivity analyses are presented in Online Data Supplement 5. When CM was defined using a diagnosis of acute myocardial infarction alone, the association between CM and in-hospital mortality remained consistent (adjusted HR, 1.82; 95% CI, 1.60-2.07). Similarly, in the analysis restricted to patients without interhospital transfer, CM continued to be associated with higher in-hospital mortality (adjusted HR, 1.72; 95% CI, 1.48-2.00). Associations with selected resource utilization outcomes were directionally consistent across sensitivity analyses.

## Discussion

In this nationwide analysis of surgically treated ATAAD in Japan, CM was identified in 3.7% of patients and was associated with markedly higher in-hospital mortality and greater healthcare resource utilization compared to patients without CM. Patients with CM frequently required invasive therapies, including vasopressor and inotropic support, mechanical circulatory support, and prolonged mechanical ventilation, reflecting severe hemodynamic instability at ICU admission. Notably, early mortality was substantial, with a considerable proportion of deaths occurring within the first 24 hours of admission and during the ICU stay. Importantly, despite substantial advances in surgical techniques and perioperative care that have improved outcomes for ATAAD overall, mortality among patients with CM remained persistently high without a clear temporal decline. These findings indicate that ATAAD complicated by CM represents a high-risk phenotype characterized by persistently poor short-term outcomes and substantial intensive care requirements.

A considerable proportion of patients with traditional risk factors for aortic dissection, such as male sex, obesity, and smoking status, were identified in this study.^20^^,^^21^ In contrast to previous reports, the younger age of patients in this cohort may have contributed to the lower prevalence of hypertension and hyperlipidemia.^21^ Notably, CM was observed more frequently in patients with a history of CABG or PCI. Although CM occurs when the dissection extends into the coronary arteries, patients with preexisting coronary artery disease may be more susceptible to compromised coronary blood flow, even with the same degree of dissection progression.^23^ Consistent with the findings of previous reports, a substantial number of patients presented with impaired consciousness, cardiac arrest, and cardiogenic shock and required catecholamine support.^24^ Consequently, the proportion of patients referred from other hospitals was significantly lower. In this context, overall improvements in ATAAD care might not translate into improved survival when profound myocardial compromise and hemodynamic collapse occur early in the clinical course.

As show in Figure 2, the mortality rate among patients with ATAAD without CM decreased over time; nevertheless, no such decline was observed in those with CM. Several mechanisms may explain the observed temporal improvement in patients without CM. Advances in emergency diagnostic imaging, broader availability of computed tomography, refinement of surgical techniques, improved myocardial protection strategies, and progress in perioperative and ICU management may have collectively contributed to improved survival. In contrast, these benefits might not fully extend to patients with CM. CM is frequently accompanied by profound myocardial injury, cardiogenic shock, and severe hemodynamic instability at presentation, conditions that may attenuate the impact of system-level or technical advances achieved in ATAAD care. Although overall mortality from aortic dissection has improved, patients with CM continue to require substantially greater perioperative mechanical circulatory support and more frequent catecholamine use compared with those without CM. In this context, severe myocardial injury and hemodynamic instability frequently coexist with early mortality in this population, in addition to the underlying aortic pathology.

Taken together, these observations suggest that the clinical course of ATAAD complicated by CM is influenced not solely by the severity of the aortic pathology itself but rather by the extent of concomitant myocardial injury and severe circulatory instability observed during the early postoperative ICU phase. In this context, the frequent need for multiple invasive interventions and the concentration of deaths within the first 24 hours and during ICU LOS highlight the severity of circulatory failure in this population and underscore the challenges faced during the early postoperative and critical care phases.

The mortality rate of our patients with CM was approximately 3.5 times higher than that in patients without CM, consistent with the results of previous studies.^6^^,^^9^^,^^10^ Importantly, it has been estimated that up to 40% of ATAAD patients die before reaching the hospital^25^; even among those in our cohort who underwent surgery, 6% died within 24 hours of admission, and 33% died during hospitalization. Multivariable analysis confirmed that CM was associated with higher in-hospital mortality after adjustment for clinically relevant covariates, supporting prior findings.^5^^,^^11^ Given the observational design and potential residual confounding, these results should be interpreted as associations rather than as causal effects.

Regarding healthcare resource use, patients without CM incurred hospitalization costs comparable to those reported in previous studies,^16^ whereas individuals with CM incurred significantly higher costs, with a difference of approximately 10,000 USD. The median hospital LOS was 14 days shorter in patients with CM compared to those without CM, likely reflecting increased in-hospital mortality in this group. The augmented costs in this group likely reflect the requirement for more intensive interventions, including mechanical circulatory support, nitric oxide therapy, and dialysis. These cost differences should be interpreted as reflecting greater intensity of care rather than differences in economic efficiency or treatment value. In Japan, the number of older adult patients with ATAAD is expected to increase, underscoring the importance of appropriately allocating limited medical resources for the management of ATAAD complicated by CM.

As shown in Online Data Supplement 4, the use of CAG and PCI on the day of admission varied over time, without a consistent pattern. In our previous nationwide analysis, early CAG was not associated with lower mortality, whereas a recent multicenter study suggested potential associations between early PCI and outcomes in selected patients.^12^^,^^22^ This discrepancy likely reflects differences in how patients who underwent CAG without PCI were classified, suggesting heterogeneity in contemporary management practices among patients with CM. Further studies integrating detailed anatomic, hemodynamic, and physiologic information are warranted to better characterize CM mechanisms and contemporary management in ATAAD, given the persistently high mortality observed over time.

This study has some limitations. First, the DPC database structure does not allow determination of the temporal sequence between diagnostic confirmation of ATAAD and procedures such as CAG or PCI, and thus we were unable to assess whether these procedures were performed before or after ATAAD diagnosis. Diagnostic delay may influence outcomes and should be considered when interpreting these findings. Second, patients with ATAAD and CM who died before hospital arrival or surgery were excluded from this analysis. Similarly, individuals who experienced cardiac arrest due to ATAAD before admission also were excluded, which may have led to underestimation of true mortality rates. Selection and survivorship bias should be considered when interpreting age differences. Patients with severe CM, particularly older individuals, may die before hospital arrival or may not be selected for surgery. Because patients who died before hospital arrival were not captured in the database, the true mortality of ATAAD with or without CM may be underestimated. Additionally, variability in institutional diagnostic and management practices could not be fully evaluated using administrative data. Third, CM was defined using administrative codes and procedures, and the possibility of misclassification cannot be excluded. We lacked electrocardiographic findings, cardiac biomarker values, imaging details, coronary anatomy, and procedural indications, and thus could not distinguish dynamic from static coronary compromise or determine the timing of myocardial infarction relative to surgery. Detailed surgical techniques (eg, ostial repair, coronary reconstruction, or central repair strategies) were not captured in the administrative database. Only codes for such procedures as CABG were available, precluding more granular analysis of operative management. Fourth, there is a possibility that a small number of patients who developed iatrogenic ATAAD during CAG or PCI were included,^26^, ^27^, ^28^ although given the very low incidence of such cases and the even smaller proportion of cases requiring surgical repair, any impact on our overall findings is likely minimal. Fifth, the DPC database does not provide reliable information on cause-specific mortality and detailed anatomic information on aortic dissection (eg, arch, descending, or abdominal involvement), and consequently, we were unable to distinguish between cardiac and noncardiac causes of death and could not evaluate anatomic differences between groups. Although the results of subgroup analyses according to coronary procedures are presented, these findings should be interpreted as descriptive rather than causal, owing to the substantial risk of confounding by indication. Finally, this study might not have fully distinguished between preoperative CM and myocardial infarction occurring after surgery, and a small proportion of patients diagnosed with postoperative myocardial infarction may have been misclassified.^29^

## Conclusions

In our study cohort, CM in patients with surgically treated ATAAD was associated with substantially higher in-hospital mortality and greater healthcare resource utilization, including intensive ICU-level support. While outcomes for patients without CM improved over time, mortality among those with CM remained persistently high, with a substantial proportion of deaths occurring early after admission. Further investigation using data sources with greater anatomic and physiologic detail is warranted.

### Declaration of Generative AI and AI-Assisted Technologies in the Writing Process

During the preparation of this work, Dr Fukano used ChatGPT for writing assistance. After using this service, the authors reviewed and edited the content as needed, and they take full responsibility for the content of the publication.

## Conflict of Interest Statement

The authors reported no conflicts of interest.

The *Journal* policy requires editors and reviewers to disclose conflicts of interest and to decline handling or reviewing manuscripts for which they may have a conflict of interest. The editors and reviewers of this article have no conflicts of interest.

## Figures and Tables

**Figure E1:**
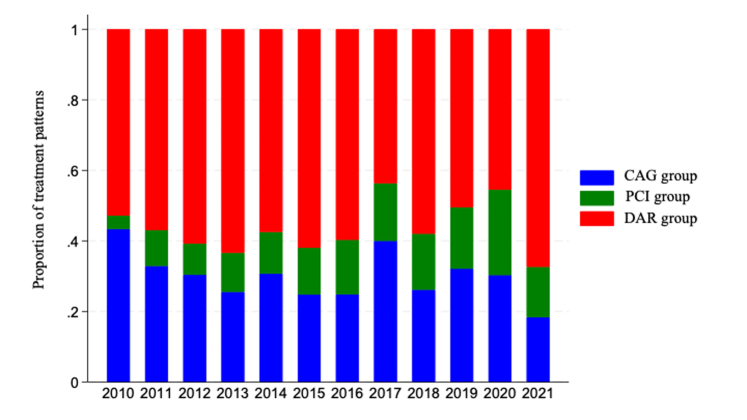
Temporal trends in management patterns among patients with acute type A aortic dissection complicated by coronary malperfusion. *CAG*, Coronary angiography; *PCI*, percutaneous coronary intervention; *DAR*, direct aortic repair.

## Supplementary Data

Table E1 Definitions of covariates.

Table E2 Components of the operational definition of coronary malperfusion.

Table E3 Detailed patient characteristics and organ-specific malperfusion of patients with acute type A aortic dissection, stratified by coronary malperfusion status.

Table E4 Sensitivity analyses for the association between coronary malperfusion and in-hospital mortality.
