# Supplementary material for: Nationwide trends in outcomes and resource utilization in surgically treated acute type A aortic dissection with coronary malperfusion
Source: JTCVS Open. 2026 Mar 19;31:101733. doi: 10.1016/j.xjon.2026.101733 (PMC13316348; doi:10.1016/j.xjon.2026.101733)
Supplement: Table E1 — Definitions of covariates. [file mmc1.pdf]

Supplementary Table S1. Definitions of Covariates

Covariates	ICD-10 codes	Definition
Postoperative myocardial infarction	I210 - 214, I219 -221, I228- 236, I249	Complications arising after admission
Mediastinitis	J985	Complications arising after admission
Postoperative shock	T811	Complications arising after admission
Hypertension	I10, I150 - 152, I159	Comorbidities present on admission
Dyslipidemia	E780 - 785	Comorbidities present on admission
Diabetic mellitus	E10, E11, E100-107, E109, E 110 - 117, E119	Comorbidities present on admission
Chronic kidney disease	N185 - N189	Comorbidities present on admission
Marfan syndrome	Q874	Comorbidities present on admission and complications arising after admission
Obstructive sleep apnea	G473	Comorbidities present on admission
Bicuspid aortic valve	Q231	Comorbidities present on admission and complications arising after admission
Loeys-Dietz syndrome	Q875	Comorbidities present on admission and complications arising after admission
Ehlers-Danlos syndrome	Q796	Comorbidities present on admission and complications arising after admission
Turner syndrome	Q96	Comorbidities present on admission and complications arising after admission
Takayasu disease	M314	Comorbidities present on admission and complications arising after admission
Behcet disease	M552	Comorbidities present on admission and complications arising after admission
Giant-cell arthritis	M316	Comorbidities present on admission and complications arising after admission
Tuberculosis	A150 - 156, A162 - 170	Comorbidities present on admission and complications arising after admission
Cardiac tamponade at admission	I319	Comorbidities present on admission
Cardiogenic shock at admission	R570	Comorbidities present on admission
Rupture at admission	I711	Comorbidities present on admission

ROSC at admission	I460	Comorbidities present on admission

The definitions were based on those used in our previous nationwide analysis of treatment strategies for ATAAD, with minor modifications for the current trend analysis. CABG: Coronary Artery Bypass Grafting, PCI: Percutaneous Coronary Intervention, ROSC: Return of Spontaneous Circulation
