# Supplementary material for: Nationwide trends in outcomes and resource utilization in surgically treated acute type A aortic dissection with coronary malperfusion
Source: JTCVS Open. 2026 Mar 19;31:101733. doi: 10.1016/j.xjon.2026.101733 (PMC13316348; doi:10.1016/j.xjon.2026.101733)
Supplement: Table E2 — Components of the operational definition of coronary malperfusion. [file mmc2.pdf]

TableS2. Components of the operational definition of coronary malperfusion

Coronary malperfusion definition	N (%)
Acute myocardial infarction diagnosis only	851 (72.9)
Coronary angiography/Percutaneous coronary intervention only	517 (44.3)
Acute myocardial infarction diagnosis and Coronary angiography/Percutaneous coronary intervention	201 (17.2)
Coronary malperfusion total	1167 (100)
