# Supplementary material for: Nationwide trends in outcomes and resource utilization in surgically treated acute type A aortic dissection with coronary malperfusion
Source: JTCVS Open. 2026 Mar 19;31:101733. doi: 10.1016/j.xjon.2026.101733 (PMC13316348; doi:10.1016/j.xjon.2026.101733)
Supplement: Table E3 — Detailed patient characteristics and organ-specific malperfusion of patients with acute type A aortic dissection, stratified by coronary malperfusion status. [file mmc3.pdf]

Supplementary Table S3. Detailed patient characteristics and organ-specific malperfusion of patients with acute type A aortic dissection, stratified by coronary malperfusion status

	Total N=31,522	No Coronary malperfusion N=30,355	Coronary malperfusion N=1,167	p value
Year, n(%)				
2010	1,139 (3.6%)	1,086 (3.6%)	53 (4.5%)	0.63
2011	1,819 (5.8%)	1,740 (5.7%)	79 (6.8%)	
2012	2,132 (6.8%)	2,053 (6.8%)	79 (6.8%)	
2013	2,304 (7.3%)	2,214 (7.3%)	90 (7.7%)	
2014	2,873 (9.1%)	2,772 (9.1%)	101 (8.7%)	
2015	3,049 (9.7%)	2,936 (9.7%)	113 (9.7%)	
2016	3,193 (10.1%)	3,064 (10.1%)	129 (11.1%)	
2017	3,177 (10.1%)	3,067 (10.1%)	110 (9.4%)	
2018	3,058 (9.7%)	2,951 (9.7%)	107 (9.2%)	
2019	2,982 (9.5%)	2,873 (9.5%)	109 (9.3%)	
2020	2,907 (9.2%)	2,808 (9.3%)	99 (8.5%)	
2021	2,889 (9.2%)	2,791 (9.2%)	98 (8.4%)	
Month, n(%)				
January	3,493 (11.1%)	3,373 (11.1%)	120 (10.3%)	0.053
February	2,994 (9.5%)	2,892 (9.5%)	102 (8.7%)	
March	2,874 (9.1%)	2,781 (9.2%)	93 (8.0%)	
April	2,637 (8.4%)	2,550 (8.4%)	87 (7.5%)	
May	2,263 (7.2%)	2,156 (7.1%)	107 (9.2%)	
June	1,955 (6.2%)	1,888 (6.2%)	67 (5.7%)	
July	1,676 (5.3%)	1,623 (5.3%)	53 (4.5%)	
August	1,691 (5.4%)	1,624 (5.4%)	67 (5.7%)	
September	2,095 (6.6%)	2,002 (6.6%)	93 (8.0%)	
October,	2,892 (9.2%)	2,779 (9.2%)	113 (9.7%)	
November	3,372 (10.7%)	3,231 (10.6%)	141 (12.1%)	

December	3,580 (11.4%)	3,456 (11.4%)	124 (10.6%)	
History of chemotherapy, n (%)	100 (0.3%)	96 (0.3%)	4 (0.3%)	0.87
Physical function at admission, n (%)				
Severe to moderate dependence (BI 0–60)	24,483 (77.7%)	23,540 (77.5%)	943 (80.8%)	0.031
Mild dependence (BI 61–99)	565 (1.8%)	548 (1.8%)	17 (1.5%)	
Complete independence (BI 100)	6,474 (20.5%)	6,267 (20.6%)	207 (17.7%)	
Past Medical history, n (%)				
Hypertension	19,131 (60.7%)	18,594 (61.3%)	537 (46.0%)	<0.001
Dyslipidemia	4,842 (15.4%)	4,704 (15.5%)	138 (11.8%)	<0.001
Diabetic mellitus	2,844 (9.0%)	2,743 (9.0%)	101 (8.7%)	0.66
Chronic kidney disease	905 (2.9%)	884 (2.9%)	21 (1.8%)	0.025
Bicuspid aortic valve	40 (0.1%)	40 (0.1%)	0 (0.0%)	0.21
Marfan syndrome	204 (0.6%)	195 (0.6%)	9 (0.8%)	0.59
Loeys-Dietz syndrome	2 (0.0%)	2 (0.0%)	0 (0.0%)	0.78
Ehlers-Danlos syndrome	1 (0.0%)	1 (0.0%)	0 (0.0%)	0.84
Turner syndrome	8 (0.0%)	8 (0.0%)	0 (0.0%)	0.58
Current pregnancy	6 (0.0%)	5 (0.0%)	1 (0.1%)	0.24
Obstructive sleep apnea	100 (0.3%)	99 (0.3%)	1 (0.1%)	0.15
Takayasu disease	8 (0.0%)	8 (0.0%)	0 (0.0%)	0.58
Behçet disease	9 (0.0%)	9 (0.0%)	0 (0.0%)	0.56
Giant-cell arthritis	9 (0.0%)	9 (0.0%)	0 (0.0%)	0.56
Tuberculosis	11 (0.0%)	10 (0.0%)	1 (0.1%)	0.34
Past history of valve surgery	53 (0.2%)	51 (0.2%)	2 (0.2%)	0.98
Past history of CABG	20 (0.1%)	17 (0.1%)	3 (0.3%)	0.007
Past history of PCI	7 (0.0%)	5 (0.0%)	2 (0.2%)	<0.001
Malperfusions, n (%)				
Cerebral malperfusion	1,569 (5.0%)	1,506 (5.0%)	63 (5.4%)	0.50
Mesenteric malperfusion	488 (1.5%)	466 (1.5%)	22 (1.9%)	0.34

Lower limb malperfusion	155 (0.5%)	152 (0.5%)	3 (0.3%)	0.24
Renal malperfusion	831 (2.6%)	786 (2.6%)	45 (3.9%)	0.008
Emergency center, n (%)	5,371 (17.0%)	5,158 (17.0%)	213 (18.3%)	0.26
Admission ward, n (%)				0.28
ICU	21,289 (67.5%)	20,497 (67.5%)	792 (67.9%)	
HCU	5,808 (18.4%)	5,610 (18.5%)	198 (17.0%)	
Admission at night or on a weekend, n (%)	4,823 (15.3%)	4,647 (15.3%)	176 (15.1%)	0.83
Ambulance use, n (%)	26,856 (85.2%)	25,843 (85.1%)	1,013 (86.8%)	0.12
Surgical management, n (%)				
CABG	1,502 (4.8%)	1,007 (3.3%)	495 (42.4%)	<0.001
AAR + AVR	1,630 (5.2%)	1,536 (5.1%)	94 (8.1%)	<0.001
Bentall	1,071 (3.4%)	978 (3.2%)	93 (8.0%)	<0.001
David Yacoub	224 (0.7%)	205 (0.7%)	19 (1.6%)	<0.001
Other AAR	16,677 (52.9%)	16,150 (53.2%)	527 (45.2%)	<0.001
AAR + TAR + AVR	879 (2.8%)	835 (2.8%)	44 (3.8%)	0.038
Bentall + TAR	530 (1.7%)	490 (1.6%)	40 (3.4%)	<0.001
David Yacoub + TAR	131 (0.4%)	125 (0.4%)	6 (0.5%)	0.59
Other AAR + TAR	6,416 (20.4%)	6,185 (20.4%)	231 (19.8%)	0.63
TAR	4,052 (12.9%)	3,933 (13.0%)	119 (10.2%)	0.006
TEVAR	151 (0.5%)	150 (0.5%)	1 (0.1%)	0.047
Volatile anesthesia, n(%)	25,632 (81.3%)	24,715 (81.4%)	917 (78.6%)	0.015

BI, Barthel index; ICU, intensive care unit; HCU, high care unit; ER, emergency room; PCI, percutaneous coronary intervention; CABG, coronary artery bypass grafting; AAR, ascending aortic replacement; AVR, aortic valve replacement; TAR, total arch replacement; TEVAR, thoracic endovascular aortic repair; SD, standard deviation; IQR, interquartile range
