# Supplementary material for: Nationwide trends in outcomes and resource utilization in surgically treated acute type A aortic dissection with coronary malperfusion
Source: JTCVS Open. 2026 Mar 19;31:101733. doi: 10.1016/j.xjon.2026.101733 (PMC13316348; doi:10.1016/j.xjon.2026.101733)
Supplement: Table E4 — Sensitivity analyses for the association between coronary malperfusion and in-hospital mortality. [file mmc4.pdf]

Supplementary Table S4. Sensitivity analyses for the association between coronary malperfusion and in-hospital mortality

Analysis	CM definition	CM, n(%)	Adjusted HR for in-hospital mortality	Total hospitalization cost difference, USD
Primary analysis	AMI diagnosis and/or Day0 CAG/PCI	1167 (3.7%)	1.80 (1.58 to 2.06)	7,173 (5,205 to 9,141)
Sensitivity analysis 1	AMI diagnosis only	851 (2.7%)	1.82 (1.60 to 2.07)	11,924 (10,191 to 13,656)
Sensitivity analysis 2	No interhospital transfer	663 (2.1%)	1.72 (1.48 to 2.00)	7,173 (5,205 to 9,141)

Sensitivity analysis 1 was performed using a more specific definition of coronary malperfusion, restricted to patients with a diagnosis of acute myocardial infarction only, to assess the robustness of the association while minimizing potential misclassification related to procedural coding. Sensitivity analysis 2 was conducted after excluding patients who underwent interhospital transfer, in order to reduce potential bias related to pre-admission management, referral patterns, and treatment delays that could influence early outcomes and resource utilization.

CM, coronary malperfusion; AMI, acute myocardial infarction; CAG, coronary angiography; PCI, percutaneous coronary intervention; HR, hazard ratio; USD, United States dollar.
